# Optimizing patient triage on the waiting list for transcatheter aortic valve replacement: the clinical utility of the cardiac damage staging system

**DOI:** 10.1093/ehjqcco/qcag001

**Published:** 2026-03-03

**Authors:** Karen Boyer, Adrien Carmona, Francois Severac, Antonin Trimaille, Kévin Roulot, Shinnosuke Kikuchi, Marion Kibler, Patrick Ohlmann, Benjamin Marchandot, Olivier Morel

**Affiliations:** Department of Cardiology, University Hospital of Strasbourg, 1 place de l'hôpital, 67000 Strasbourg, France; GERCA, Groupe pour l’Enseignement et la Recherche Cardiovasculaire en Alsace, 1 place de l'hôpital, 67000 Strasbourg, France; Department of Cardiology, University Hospital of Strasbourg, 1 place de l'hôpital, 67000 Strasbourg, France; GERCA, Groupe pour l’Enseignement et la Recherche Cardiovasculaire en Alsace, 1 place de l'hôpital, 67000 Strasbourg, France; Biostatistics Department, University Hospital of Strasbourg, 1 place de l'hôpital, 67000 Strasbourg, France; Department of Cardiology, University Hospital of Strasbourg, 1 place de l'hôpital, 67000 Strasbourg, France; GERCA, Groupe pour l’Enseignement et la Recherche Cardiovasculaire en Alsace, 1 place de l'hôpital, 67000 Strasbourg, France; Research Unit—UR3074, Translational Cardiovascular Medicine, University of Strasbourg, 1 place de l'hôpital, 67000 Strasbourg, France; Department of Cardiology, University Hospital of Strasbourg, 1 place de l'hôpital, 67000 Strasbourg, France; GERCA, Groupe pour l’Enseignement et la Recherche Cardiovasculaire en Alsace, 1 place de l'hôpital, 67000 Strasbourg, France; Department of Cardiology, University Hospital of Strasbourg, 1 place de l'hôpital, 67000 Strasbourg, France; GERCA, Groupe pour l’Enseignement et la Recherche Cardiovasculaire en Alsace, 1 place de l'hôpital, 67000 Strasbourg, France; Research Unit—UR3074, Translational Cardiovascular Medicine, University of Strasbourg, 1 place de l'hôpital, 67000 Strasbourg, France; Department of Cardiology, University Hospital of Strasbourg, 1 place de l'hôpital, 67000 Strasbourg, France; GERCA, Groupe pour l’Enseignement et la Recherche Cardiovasculaire en Alsace, 1 place de l'hôpital, 67000 Strasbourg, France; Department of Cardiology, University Hospital of Strasbourg, 1 place de l'hôpital, 67000 Strasbourg, France; GERCA, Groupe pour l’Enseignement et la Recherche Cardiovasculaire en Alsace, 1 place de l'hôpital, 67000 Strasbourg, France; Department of Cardiology, University Hospital of Strasbourg, 1 place de l'hôpital, 67000 Strasbourg, France; GERCA, Groupe pour l’Enseignement et la Recherche Cardiovasculaire en Alsace, 1 place de l'hôpital, 67000 Strasbourg, France; Research Unit—UR3074, Translational Cardiovascular Medicine, University of Strasbourg, 1 place de l'hôpital, 67000 Strasbourg, France; Department of Cardiology, University Hospital of Strasbourg, 1 place de l'hôpital, 67000 Strasbourg, France; GERCA, Groupe pour l’Enseignement et la Recherche Cardiovasculaire en Alsace, 1 place de l'hôpital, 67000 Strasbourg, France; Research Unit—UR3074, Translational Cardiovascular Medicine, University of Strasbourg, 1 place de l'hôpital, 67000 Strasbourg, France; Hanoï Medical University, 1 P. Tôn Thất Tùng, Kim Liên, Đống Đa, Hanoï, Vietnam

**Keywords:** Aortic stenosis, Waiting list, TAVI, Heart failure, Mortality

## Abstract

**Aims:**

Access to transcatheter aortic valve replacement (TAVR) is generally prioritized for symptomatic patients presenting with severe heart failure, syncope, angina, and decrease in left ventricular ejection fraction <40%. However, a substantial number of patients die while awaiting TAVR. In response to rising demand driven by an ageing population, it is essential to stratify interventions, as proposed by Généreux’s Aortic Stenosis staging.

**Methods and results:**

We conducted a retrospective, single-center, longitudinal study in patients referred for TAVR. We specifically analysed the characteristics of 98 patients who died while on the TAVR waiting list. The primary outcome was all-cause mortality from the time of a patient’s inclusion on the TAVR waiting list or non-proceeding to TAVR. The overall proportion of deceased patients was 8.4% [95% confidence interval (CI), 6.9–10.1] and 4% when excluding deaths occurring after 2019. By 3 months, 61.2% of patients had undergone TAVR, and this increased to 85.4% at 6 months. Deaths occurred in 6.3% of patients by 3 months and 7.8% by 6 months. We notice that 90% of deaths took place within the first 3 months. Multivariable analysis identified several variables independently associated with mortality on the TAVR waiting list: prior myocardial infarction [subdistribution hazard ratio (sHR), 1.87 (95% CI, 1.12–3.13); *P* = 0.017] and pulmonary artery systolic pressure > 60 mmHg [sHR, 2.37 (95% CI, 1.36–4.12); *P* = 0.002]. Généreux stages 3 and 4 showed a strong association with waiting-list mortality, more than doubling the risk of death [sHR, 2.12 (95% CI, 1.33–3.38); *P* = 0.002].

**Conclusion:**

These findings suggest that TAVR candidates at Généreux stages 3 and 4 should receive higher priority on the waiting list.

## Graphical Abstract

**Graphical Abstract:**
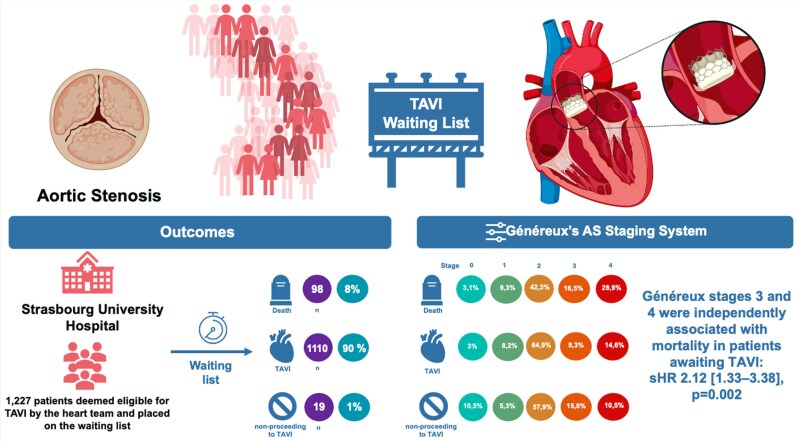

Key Learning Points What is already known:

The natural history of severe aortic stenosis is poor: many patients die while on the waiting list for transcatheter aortic valve implantation. Established risk factors for pre-procedural mortality include reduced left ventricular ejection fraction, syncope, and unstable angina. Whether these deaths are due solely to system delays or to additional, unrecognized factors remains uncertain.

What this study adds:

Our study shows that patients in stages 3 and 4 of the Généreux classification have a significantly higher risk of death while awaiting transcatheter aortic valve implantation (TAVI). Right ventricular dysfunction and elevated pulmonary artery pressures also emerge as independent predictors of pre-TAVI mortality.

## Introduction

Aortic stenosis (AS) is the most common valvular disease in industrialized countries^1,2^ with an estimated 180 000 potential transcatheter aortic valve replacement (TAVR) procedures in the European Union and Northern America and 270 000 TAVR projected if indication to TAVR expend to low risk.^3^ As the ageing population grows, the demand for TAVR has surged, leading to the development of extensive waiting lists. Despite the well-established benefits of TAVR for symptomatic severe AS, prolonged waiting times have been associated with adverse clinical outcomes,^4-6^ increased healthcare costs,^7^ and ethical dilemmas for caregivers.^8^

Traditionally, the medical and surgical staff’s role was limited to selecting candidates for surgical aortic valve replacement (SAVR) or TAVR. However, as waiting lists expand, the decision-makers must now also assess urgency, with triage, risk stratification, and individualized care emerging as key elements in the selection and procedural planning for TAVR. Access to TAVR is prioritized for symptomatic patients exhibiting signs of severe heart failure, syncope, or angina.^9,10^

Recent data from large Danish and Swedish national registries demonstrate that, in the contemporary era, long-term mortality after transcatheter aortic valve implantation (TAVI) is comparable to that of an age- and sex-matched general population after adjustment for comorbidities, except in highly comorbid patients.^11,12^ These findings suggest that TAVI itself is not associated with excess long-term mortality and that the observed survival disadvantage in patients with severe AS is likely driven predominantly by the pre-interventional period. In this context, waiting-list time appears to be a major determinant of mortality, underscoring the need for optimized waiting-list management, particularly in younger or non-comorbid patients in whom the expected benefit of TAVI is greatest.

This evolving challenge has driven the need for structured procedural planning and individualized care, yet it has also led to an implicit acceptance of treatment delays and increased mortality among waitlisted patients. Given the increasing burden of AS and the growing demand for TAVR, reliable risk stratification tools are imperative to differentiate patients who can safely wait from those requiring urgent intervention. However, patient triage and the need for reassessment while on the TAVR waiting list remain an underdeveloped area studied.

The AS staging classification proposed by Généreux et al. offers an objective framework to characterize the extent of extra-valvular cardiac damage associated with AS.^13^ This staging system has demonstrated significant prognostic implications for clinical outcomes following aortic valve replacement, yet its application in risk stratification of patients on the TAVR waitlist has not been explored.

In this study, we aim to evaluate the utility of AS staging for prioritizing patients on the TAVR waitlist, providing an evidence-based approach to triage in response to the growing public health challenge posed by severe AS.

## Methods

### Study design and participants

We conducted a retrospective, monocentric longitudinal study including all patients referred for TAVR between February 2010 and March 2024 at our institution (Strasbourg University Hospital, Strasbourg, France). Patients on the waiting list were those who had been evaluated and deemed eligible for TAVR by the medical and surgical staff but had not yet undergone the procedure, awaiting scheduling and intervention. Data from 1227 patients eligible for TAVR enabled the characterization of demographic parameters, medical history, echocardiographic, angiographic, biological, and imaging data. Patients were further classified according to the Généreux staging system, based on the presence or absence of extra-valvular cardiac damage detected by echocardiography. This staging system includes five independent, non-cumulative stages (0–4)^13^:

- *Stage 0*: No extra-valvular cardiac involvement.
- *Stage 1*: Left ventricular involvement, with left ventricular hypertrophy defined by a left ventricular mass strictly greater than 95 g/m^2^ in women and 115 g/m^2^ in men; severe diastolic dysfunction defined by an *E*/*e*′ ratio >14; or impaired systolic function with an left ventricular ejection fraction (LVEF) <50%.
- *Stage 2*: Mitral valve involvement with at least grade 3/4 mitral regurgitation (i.e. moderate), defined by an effective regurgitant orifice area (EROA) > 30 mm^2^ and a regurgitant volume (RV) > 45 mL; a dilated left atrium; or the presence of atrial fibrillation.
- *Stage 3*: Pulmonary hypertension defined by a systolic pulmonary artery pressure (SPAP) > 60 mmHg, or tricuspid valve involvement with moderate (EROA >20 mm^2^ or RV >30 mL) to severe tricuspid regurgitation.
- *Stage 4*: Right ventricular involvement defined by a right ventricular fractional shortening strictly <35%.

Patient data were censored at the time of the TAVR procedure, defined as the moment of valve implantation, or if the patient died before the index procedure.

All patients signed informed consent before the procedure and agreed to the anonymous processing of their data (France 2 and France TAVI Registries). The study protocol was developed in accordance with the Declaration of Helsinki and was approved by the France 2 study: 911 262.

### Outcomes

The primary endpoint was all-cause mortality from the time of a patient’s inclusion on the TAVR waiting list or non-proceeding to TAVR. Non-proceeding to TAVR included three categories: (1) treatment ineligibility, defined as patients deemed unsuitable for TAVR by the medical and surgical staff due to excessive procedural risk (e.g. cardiogenic shock, advanced dementia, or sepsis); (2) treatment deferral, referring to patients with a temporary contraindication leading to procedural postponement due to clinical deterioration; and (3) voluntary postponement, in which patients personally requested to delay the intervention.

The secondary objective was to identify clinical, biological, and echocardiographic predictors of mortality among patients on the waiting list, comparing them to those who underwent percutaneous valve replacement using the Généreux classification.^13^

### Statistical analyses

Continuous variables are presented as medians with interquartile ranges. Categorical variables are presented as counts and percentages. The cumulative incidence of death on the waiting list was estimated using a competing risks approach. Time to event was defined as the period between the date of inclusion on the waiting list and the date of death, considering TAVI, or non-proceeding to TAVR, as competing risks. The Fine and Gray model was used to identify baseline characteristics associated with the cumulative incidence function of death. A multivariate model was constructed including variables with clinical relevance and/or a *P*-value <0.1 in univariate analysis. To avoid collinearity between variables [LVEF <40%, SPAP >60 mmHg, right ventricular shortening fraction (RVSF) <35%, and Généreux stage 3 or 4], two distinct multivariate analyses were performed. Results are presented as subdistribution hazard ratios (sHRs) with 95% CIs. A *P*-value <0.05 was considered statistically significant. Given the retrospective design of the study and the low proportion of missing data for key variables (the variable with the most missing data is SPAP, with 13.2%), no imputation was used and complete cases analysis was performed. Analyses were performed using R software version 4.1.1.

## Results

### Population

**Figure 1:**
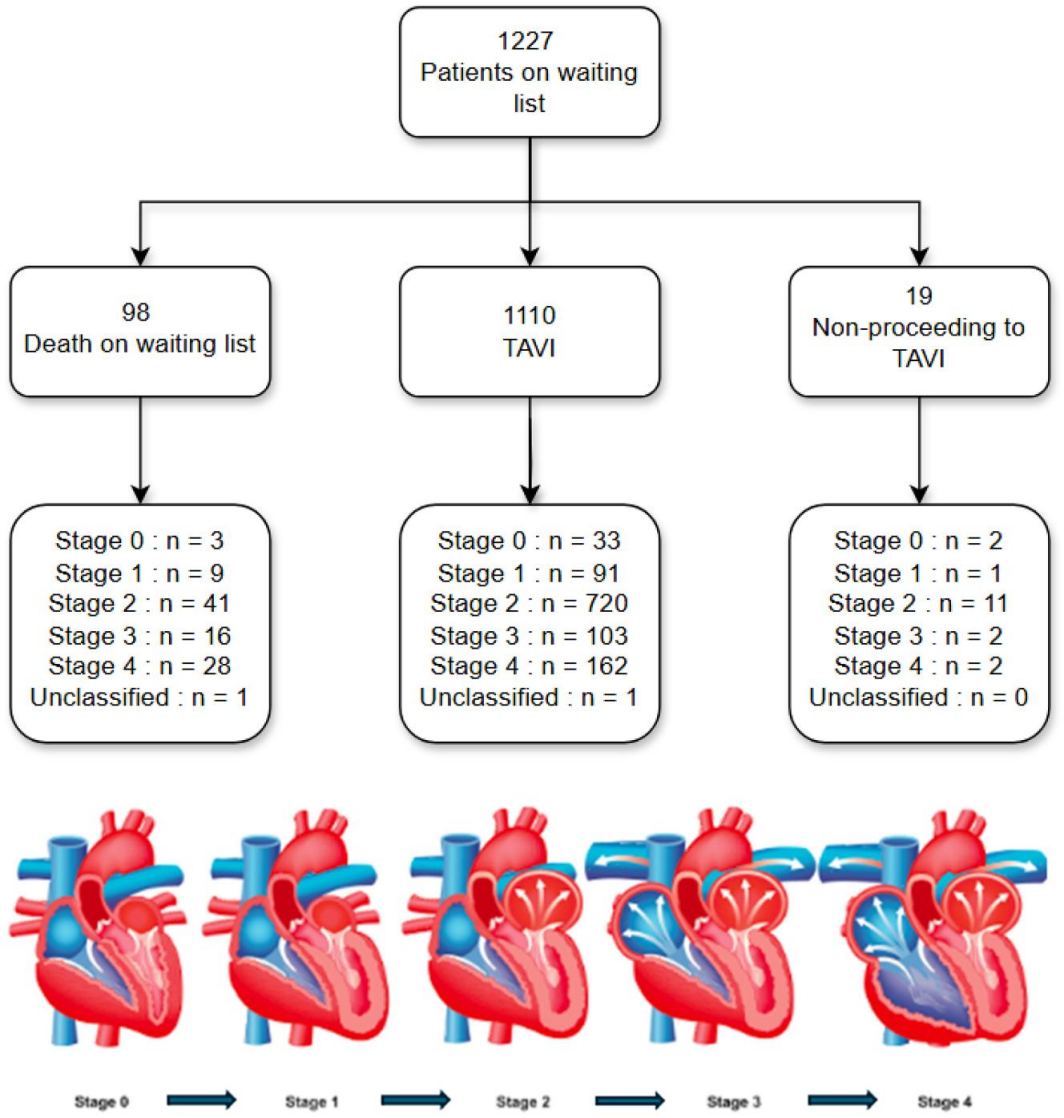
Flow chart of the study. Among 1227 patients on the transcatheter aortique valve implantation waiting list, 1110 underwent the procedure, 98 died while waiting, and 19 were classified non-proceeding to transcatheter aortique valve implantation. The study population was categorized into three groups and stratified according to the Généreux aortic valve staging system.^13^

A total of 1227 patients deemed eligible for TAVR by the medical and surgical staff and placed on the waiting list, 1110 underwent the procedure, 98 died while waiting, and 19 were classified as non-proceeding to TAVR (*Figure 1*). Reasons for not proceeding with TAVR included: ineligible for treatment (*n* = 2), treatment deferral (*n* = 2), and voluntary postponement (*n* = 15).

### Clinical characteristics

Patients who died while on the TAVR waiting list were more likely to be men (*P* = 0.015) and had a higher burden of cardiovascular risk factors, including diabetes (*P* = 0.005), dyslipidemia (*P* = 0.002), hypertension (*P* = 0.088), and dialysis dependence (*P* = 0.006), compared to those who underwent TAVR (*Table 1*). These patients also exhibited a greater ischaemic burden, with higher rates of prior myocardial infarction (*P* < 0.001), peripheral arterial disease (*P* = 0.078), and chronic kidney disease (53.6% vs. 19%, *P* < 0.001). Although not statistically significant, asymptomatic presentation was more frequent in the deceased group compared to those who underwent TAVR (8.2% vs. 4.3%, *P* = 0.124; *Table 1*).

**Table 1:** Baseline characteristics

	No. (%)			
	Deaths	TAVI	Non-proceeding to TAVR	*P* (between death and TAVR)
*n*	98	1110	19	
**Clinical characteristics**				
Median age (IQR)	83.00 [77.00, 87.00]	84.00 [80.00, 87.00]	83.00 [81.00, 87.50]	0.159
Male sex %	58 (59.2)	509 (45.9)	4 (21.1)	0.015
Median BMI (IQR)	26.57 [23.63, 29.10]	26.38 [23.00, 29.87]	25.05 [21.38, 26.60]	0.949
Active smoking (%)	4 (4.1)	40 (3.6)	1 (5.3)	0.775
HT (%)	87 (89.7)	920 (82.9)	16 (84.2)	0.088
Diabetes (%)	45 (46.4)	355 (32.0)	4 (21.1)	0.005
Dyslipidemia (%)	72 (75.0)	658 (59.3)	13 (68.4)	0.002
COPD (%)	15 (15.5)	150 (13.5)	2 (10.5)	0.643
Myocardial infarction (%)	30 (30.9)	139 (12.5)	4 (21.1)	<0.001
PCA (%)	39 (40.2)	381 (34.3)	6 (31.6)	0.267
CABG (%)	8 (8.2)	122 (11.0)	1 (5.3)	0.496
Stroke (%)	15 (15.5)	164 (14.8)	4 (21.1)	0.881
Creatinine > 150 µmol/L (%)	52 (53.6)	211 (19.0)	5 (26.3)	<0.001
Dialysis (%)	9 (9.3)	35 (3.2)	1 (5.3)	0.006
AF (%)	56 (57.7)	505 (45.5)	8 (42.1)	0.025
PAD (%)	34 (35.1)	295 (26.6)	2 (10.5)	0.075
HF (%)	50 (52.1)	487 (43.9)	12 (63.2)	0.134
**Symptoms**				
Dyspnoea (%)	80 (82.5)	995 (89.8)	17 (89.5)	0.038
Angina (%)	18 (18.6)	127 (11.5)	2 (10.5)	0.05
Syncope (%)	11 (11.3)	123 (11.1)	2 (10.5)	0.867
Asymptomatics (%)	8 (8.2)	48 (4.3)	1 (5.3)	0.124
NYHA (%)				0.07
1	18 (18.6)	112 (10.1)	2 (10.5)	
2	33 (34.0)	376 (33.9)	10 (52.6)	
3	35 (36.1)	498 (44.9)	5 (26.3)	
4	11 (11.3)	122 (11.0)	2 (10.5)	
**Généreux staging (%)**				<0.001
0	3 (3.1)	33 (3.0)	2 (10.5)	
1	9 (9.3)	91 (8.2)	1 (5.3)	
2	41 (42.3)	720 (64.9)	11 (57.9)	
3	16 (16.5)	103 (9.3)	3 (15.8)	

Data represented by medians with IQR or percentages (%) with *n*, number of subjects; AF, atrial fibrillation; BMI, body mass index; CABG, coronary artery bypass graft; COPD, chronic obstructive pulmonary disease; HF, heart failure; HT, hypertension; IQR, interquartile range; NYHA, New York Heart Association; PAD, peripheral arterial disease; PCA, percutaneous coronary angioplasty; TAVI, transcatheter aortic valve implantation; TAVR, transcatheter aortic valve replacement.

### Généreux’s aortic stenosis staging system

Regarding AS severity, patients had an average Doppler velocity index of 0.20, with an indexed valvular area <0.60 cm^2^/m^2^ in both the deceased and TAVR groups, compared to 0.73 cm^2^/m^2^ in the non-proceeding to TAVR group. Concerning right ventricular function, the deceased group had 30.5% with altered RVSF (<35%), compared to 14.9% in the TAVR group and 10.5% in the excluded group (see Supplementary material online, *Table S1*). In the deceased group, the distribution of cardiac damage stages was as follows: 3.1% at stage 0, 9.3% at stage 1, 42.3% at stage 2, 16.5% at stage 3, and 28.9% at stage 4. In comparison, the TAVR group had 3.0% at stage 0, 8.2% at stage 1, 64.9% at stage 2, 9.3% at stage 3, and 14.6% at stage 4. The prevalence of stage 3 disease was similar between the deceased (16.5%) and the non-proceeding group (15.8%) but notably lower in the TAVR group (9.3%). Conversely, stage 4 disease was more prevalent in the deceased group compared to the other cohorts, underscoring the greater burden of advanced cardiac damage in this population (*Table 1*).

### Cause of death on waiting list

Of our 98 patients who died on the TAVR waiting list, 48% died of cardiovascular causes, mainly sudden death (*n* = 10) or cardiogenic shock (*n* = 30). In our cohort, 11% died of non-cardiovascular causes and 40% of unknown causes (see Supplementary material online, *Table S2*).

### Cumulative incidence analysis

**Figure 2:**
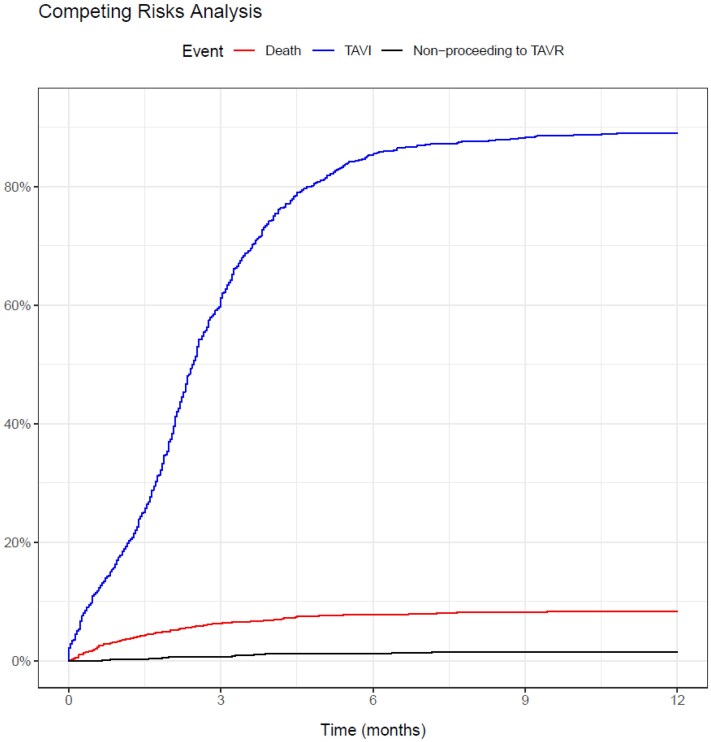
Cumulative incidence of death. The overall proportion of deceased patients was 8.4% [95% confidence interval (6.9; 10.1)]. At 3 months, 61.2% had undergone transcatheter aortique valve replacement, increasing to 85.4% at 6 months. Deaths occurred in 6.3% at 3 months, 7.8% at 6 months, and 8.3% at 12 months. Ninety percent of deaths occurred within the first 3 months. Median time to death was 1.4 months, and median time to transcatheter aortique valve implantation was 2.2 months (1.3–3.4).

The median time from inclusion in TAVR waiting list to TAVR was 2.2 months [1.3–3.4]. By 3 months, 61.2% of patients had undergone TAVR, increasing to 85.4% by 6 months. Mortality rates were 6.3% at 3 months and 7.8% at 6 months. This mortality rate is 4% when excluding patients who died after 2019. Notably, 90% of deaths occurred within the first 3 months, highlighting the early vulnerability of patients awaiting the procedure (*Figure 2*).

### Univariate analysis

Univariate analysis identified several clinical, biological, and echocardiographic parameters associated with mortality on the TAVR waiting list. Among clinical factors, male sex [sHR 1.73 (1.16–2.59), *P* = 0.007], dyslipidemia [sHR 1.96 (1.24–3.11), *P* = 0.004], diabetes [sHR 1.81 (1.22–2.70), *P* = 0.004], atrial fibrillation [sHR 1.63 (1.09–2.43), *P* = 0.018], and prior myocardial infarction [sHR 2.81 (1.83–4.30), *P* < 0.001] were associated with an increased risk of mortality. Markers of renal dysfunction, including chronic kidney disease with serum creatinine >150 μmol/L [sHR 4.53 (3.04–6.74), *P* < 0.001] and dialysis dependence [sHR 3.16 (1.61–6.21), *P* < 0.001], were also strong predictors of mortality. Being asymptomatic was not protective against waiting-list mortality [sHR 1.82 (0.89–3.72), *P* = 0.098] (*Table 2*). Among biological markers, elevated C-reactive protein (CRP) was significantly associated with increased mortality risk [sHR 3.44 (2.11–5.61), *P* < 0.001] (*Table 2*). Echocardiographic parameters linked to increased waiting-list mortality included elevated systolic pulmonary arterial pressure (SPAP) [sHR 3.00 (1.94–4.64), *P* < 0.001] and right ventricular systolic dysfunction [sHR 2.44 (1.58–3.78), *P* < 0.001]. Additional predictors included left ventricular end-diastolic diameter [sHR 1.87 (1.14–3.08), *P* = 0.013] and low cardiac output [sHR 0.73 (0.55–0.97), *P* = 0.027]. However, none of the individual Généreux stages (0, 1, 2, 3, or 4) were independently associated with waiting-list mortality. When grouped as stages 0–1, stage 2, and stages 3–4, advanced stages (3 and 4) did not reach statistical significance for predicting mortality [sHR 1.73 (0.92–3.24), *P* = 0.091] (*Table 2*).

**Table 2:** Univariate analysis: factors associated with death on the TAVR waiting list

	sHR [95% CI]	*P*
**Clinical characteristics**		
Age	0.99 [0.96–1.01]	0.328
Male sex	1.73 [1.16–2.59]	0.007
Active smoking	1.18 [0.44–3.16]	0.748
Diabetes	1.81 [1.22–2.70]	0.004
Dyslipidemia	1.96 [1.24–3.11]	0.004
Myocardial infarction	2.81 [1.83–4.30]	<0.001
PCA	1.31 [0.87–1.96]	0.194
CABG	0.76 [0.37–1.56]	0.459
Stroke	1.04 [0.60–1.81]	0.876
Creatinine (>150 μmol/L)	4.53 [3.04–6.74]	<0.001
CRF	3.16 [1.61–6.21]	<0.001
AF	1.63 [1.09–2.43]	0.018
PAD	1.48 [0.98–2.25]	0.064
HF	1.41 [0.95–2.11]	0.089
**Symptoms**		
Asymptomatic	1.82 [0.89–3.72]	0.098
Syncope	1.04 [0.55–1.96]	0.897
NYHA	Reference	
1	0.56 [0.31–0.98]	0.044
2	0.47 [0.27–0.83]	0.009
3	0.60 [0.28–1.27]	0.180
4		
**Echocardiographic parameters**		
LVEF < 40%	0.66 [0.37–1.19]	0.165
RVSF < 35%	2.44 [1.58–3.78]	<0.001
SPAP > 60 mmHg	3.00 [1.94–4.64]	<0.001
**Anticoagulant and/or antiaggregant treatment**		
Dual antiplatelet therapy	0.65 [0.38–1.12]	0.119
Clopidogrel	0.91 [0.56–1.48]	0.711
Anticoagulants	1.50 [1.00–2.24]	0.048
**Biologie**		
CRP > 4 mg/L	3.44 [2.11–5.61]	<0.001
**Aortic sténosis staging**		
0	Reference	
1	1.08 [0.30–3.90]	0.902
2	0.65 [0.21–2.06]	0.467
3	1.73 [0.51–5.81]	0.377
4	1.90 [0.59–6.10]	0.284
**Aortic Stenosis staging**		
0–1	Reference	
2	0.62 [0.33–1.16]	0.134
3–4	1.73 [0.92–3.24]	0.091

Data represented by AF, atrial fibrillation; CABG, coronary artery bypass graft; CI, confidence interval; COPD, chronic obstructive pulmonary disease; CRF, chronic renal failure; CRP, C-reactive protein; GFR, glomerular filtration rate; HF, heart failure; LVEF, left ventricular ejection fraction; NYHA, New York Heart Association classifying dyspnea from 1 to 4; PAD, peripheral arterial disease; PCA, percutaneous coronary angioplasty; RVSF, right ventricular shortening fraction; sHR, subdistribution hazards ratios; SPAP, systolic pulmonary arterial pressure; TAVR, transcatheter aortic valve replacement.

### Multivariable analysis

Two distinct multivariable models were used: one incorporating echocardiographic parameters (LVEF, RVSF, and SPAP) and another excluding echocardiography but including Généreux stages 3 and 4.

### Model 1: including echocardiographic parameters

In this model, CRP remained a strong independent predictor of waiting-list mortality [sHR 2.72 (1.64–4.51), *P* < 0.001], along with dyslipidemia [sHR 1.75 (1.03–2.97), *P* = 0.039] and prior myocardial infarction [sHR 1.87 (1.12–3.13), *P* = 0.017]. Although reduced RVSF did not reach statistical significance, it showed a strong trend [sHR 1.63 (0.97–2.75), *P* = 0.065]. SPAP >60 mmHg was independently associated with mortality [sHR 2.37 (1.36–4.12), *P* = 0.002].

### Model 2: including Généreux stages 3 and 4

In the second model, dyslipidemia [sHR 1.70 (1.03–2.81), *P* = 0.037], elevated CRP [sHR 2.68 (1.81–4.42), *P* < 0.001], renal insufficiency with creatinine >150 µmol/L [sHR 3.00 (1.81–4.99), *P* < 0.001], and prior myocardial infarction [sHR 1.79 (1.09–2.96), *P* = 0.023] remained independently associated with waiting-list mortality. Généreux stages 3 and 4 emerged as significant predictors of mortality, with more than a two-fold increased risk of death [sHR 2.12 (1.33–3.38), *P* = 0.002] (*Table 3*).

**Table 3:** Multivariate analysis

	sHR	Lower limit CI 95%	Upper limit CI 95%	*P*
**Model 1 : study of cardiac function parameters**				
Sex (male = 1)	1.26	0.77	2.04	0.36
Creatinine > 150 μmol/L	3.02	1.8	5.07	<0.001
Anticoagulation	0.91	0.39	2.09	0.82
CRP	2.72	1.64	4.51	<0.001
Dyslipidemia	1.75	1.03	2.97	0.039
Diabetes	1.25	0.78	2.01	0.36
Myocardial infarction	1.87	1.12	3.13	0.017
NYHA 2	0.71	0.34	1.47	0.36
NYHA 3 and 4	0.48	0.24	0.97	0.041
AF	1.03	0.47	2.27	0.94
RVSF < 35%	1.63	0.97	2.75	0.065
SPAP > 60 mmHg	2.37	1.36	4.12	0.002
LVEF < 40%	0.44	0.23	0.87	0.018
**Model 2 : staging study**				
Sex (male = 1)	1.17	0.73	1.88	0.52
Creatinine > 150 μmol/L	3	1.81	4.99	<0.001
Anticoagulation	0.92	0.4	2.11	0.85
CRP	2.68	1.63	4.42	< 0.001
Dyslipidemia	1.7	1.03	2.81	0.037
Diabetes	1.25	0.79	1.98	0.34
Myocardial infarction	1.79	1.09	2.96	0.023
NYHA 2	0.68	0.33	1.42	0.31
NYHA 3 and 4	0.53	0.26	1.06	0.074
AF	1.07	0.49	2.34	0.86
Staging 3 and 4	2.12	1.33	3.38	0.002

Data represented by AF, atrial fibrillation; CI, confidence interval; CRP, C-reactive protein; LVEF, left ventricular ejection fraction; NYHA, New York Heart Association classifying dyspnea from 1 to 4; RVSF, right ventricular shortening fraction; sHR, subdistribution hazards ratios; SPAP, systolic pulmonary arterial pressure.

## Discussion

In a cohort of 1227 patients deemed eligible for TAVR by the medical and surgical staff, 98 patients died while on the waiting list. The main findings of this study are as follows: (i) inflammatory burden (CRP), ischaemic burden (prior MI), and dyslipidemia were strong predictors of waiting-list mortality; (ii) elevated pulmonary artery systolic pressure (SPAP >60 mmHg) and impaired right ventricular function significantly increased mortality risk; (iii) Généreux stages 3 and 4 were independently associated with increased mortality, underscoring the prognostic significance of advanced extra-valvular cardiac damage; and (iv) median time to death was 1.4 months and median time to TAVI was 2.2 months.

These findings highlight the critical importance of timely intervention and the need for optimized risk stratification in patients awaiting TAVR.

### Généreux classification in waiting-list patients

First, investigating the Généreux classification for extra-aortic cardiac involvement in patients on the TAVI waiting list is a novel contribution. Currently, we expedite care for patients with severe symptoms, cardiogenic shock, syncope, or severely reduced LVEF. Drawing on a large patient sample, our findings validate the prognostic value of staging cardiac damage in patients with AS and support its use as a reliable tool for determining the optimal timing for patients on the waiting list. These stages emerged as an independently significant predictor of waiting-list mortality, conferring more than twice the risk of death. This result is consistent with another real-world study in symptomatic AS patients not receiving TAVI, which showed that Généreux stage 4 doubled the risk of death.^14^ In a 2020 study by E. Mara Vollema *et al.*, focusing on patients with severe symptomatic AS, Généreux staging was likewise associated with higher mortality for stages 3 and 4.^15^ These findings imply that extra-aortic cardiac involvement may be at least as critical as traditional parameters for assessing severe AS.

### Symptom status and waiting-list mortality

Second, regarding patients on the TAVI waiting list, being asymptomatic appeared to correlate with an increased risk of waiting-list mortality. One explanation is that some ‘asymptomatic’ patients may have unrecognized or unreported symptoms, introducing a possible misclassification bias. More frequent use of stress echocardiography could have identified changes in pulmonary artery pressure during exertion, which can inform the decision to proceed with TAVI.^16,17^ Moreover, the 2021 and 2025 European Society of Cardiology (ESC) guidelines broadened TAVI indications for asymptomatic patients with severe AS,^18,19^ all guided by biomarkers (brain natriuretic peptide and troponin), which may have led to a higher proportion of asymptomatic individuals on the waiting list. Because the TAVI group enrolment ended in 2019, whereas inclusion in the deceased group extended until 2024, this discrepancy in recruitment periods may partly explain the observed differences. Moreover, a number of asymptomatic patients with elevated biomarkers are likely to have more advanced disease and require earlier management.^20^

Furthermore, it is likely that a group of asymptomatic patients could have benefited from modern imaging techniques such as cardiac magnetic resonance imaging (MRI) or global longitudinal strain analysis to detect subclinical myocardial lesions and better define the actual functional status of these patients. Traditional staging may have underestimated the severity of the disease in these patients.^15,21,22^

In univariate analysis, angina seemed to increase waiting-list mortality, suggesting that these patients did not receive expedited care. Conversely, the seemingly ‘protective’ trend of LVEF <40% and severe dyspnoea (New York Heart Association III–IV) appears contradictory to routine clinical practice, where symptomatic patients typically undergo more urgent TAVI. This result does not necessarily constitute bias but instead reflects our centre’s strategy of rapid intervention for these individuals. When we excluded patients listed after May 2019, the same trend persisted: there were fewer patients with LVEF <40% in the deceased group, while more patients with LVEF <40% underwent TAVI. Currently, LVEF strongly influences treatment decisions. However, when we performed a univariate analysis excluding patients recruited after May 2019, neither dyspnoea, asymptomatic status, nor LVEF <40% were protective or unfavourable predictors. In a 2015 study, among patients who were candidates for TAVR and had not been treated, in the face of refusal or among those ultimately ineligible, those with left ventricular dysfunction had a higher risk of mortality.^23^ Future studies excluding highly symptomatic patients (e.g. severe dyspnoea) or those with markedly impaired LVEF might clarify which patient subsets truly suffer from wait times.

### Waiting times and survival

In our analysis, the median waiting time was 2.2 months, with a median of 1.4 months to death. We report an 8.4% overall mortality in this cohort since the onset of our TAVR programme (approximately 4% when excluding patients placed on the waiting list after 2019).

In a 2014 U.S. study by Malaisrie *et al.*, the median time to TAVR or SAVR was 2.9 weeks. In the first 6 weeks, 61.2% of patients underwent TAVI and 75.2% at 10 weeks.^24^ In a 2018 Canadian study by Elbaz *et al.*, mortality was 2% at 80 days.^25^ Our findings highlight that the first 3 months are critical, with increased mortality during this interval. It is important to note that, in our centre, TAVR dates are not always pre-scheduled at the time of listing, potentially prolonging the waiting interval. Among studies published after 2021, only the work by Poinsignon *et al.* (2025) in *Archives of Cardiovascular Disease* provides data directly comparable to ours, reporting similar findings from two French centres with a median waiting time of 79 days and a waitlist mortality of 5.8%.^26^ Beyond this study, no recent publication has specifically evaluated TAVI waitlist mortality as a primary endpoint with stratification by waiting time or baseline risk features. However, a 2022 study demonstrated that prolonged waiting times were associated with higher post-TAVI mortality, highlighting the prognostic implications of delays in care.^6^ Furthermore, a recent contribution from the *International Journal of Cardiology* proposed a clinical prioritization algorithm for TAVI candidates based on LVEF, transaortic gradient, and the presence of syncope, underscoring the growing interest in optimizing waitlist management.^27^ Altogether, contemporary evidence remains limited, reinforcing the relevance of our study. In France, all TAVR candidates must be discussed by a medical and surgical staff, which suggests centres should adopt a maximal waiting time policy and perhaps schedule an intervention date before the medical and surgical meeting.

### Is it time to adopt a more liberal TAVR strategy at non-cardiac sites?

Our findings suggest that a novel TAVR pathway—emphasizing local workup based on cardiac staging—could refine patient triage and optimize waiting-list management. At our university hospital, overall waiting-list mortality was 8.4%. In contrast, in the PARTNER 3 trial (503 TAVR patients), only one procedure (0.2%) was converted to surgery and two deaths (0.3%) occurred during the index hospitalization, yielding a cumulative early risk of approximately 0.6%. Similarly, in the PARTNER 2 trial (1011 TAVR patients), early mortality (defined as death during the procedure or within 3 days) was observed in 10 patients (0.9%).^28,29^

However, our 8.4% mortality rate on the waiting list spans a 14-year period, with an over-representation of deaths occurring after 2019 (0.84% per year, although the temporal distribution of deaths may be heterogeneous). Given the steep rise in TAVR procedures since 2019, we currently lack sufficient data to conclude that this rate could be reduced by half. Furthermore, any move to broaden TAVR beyond cardiac surgery centres must ensure stable complication rates; as Oettinger et al. highlight, maintaining a minimum annual TAVR volume per centre is critical for minimizing complications.^30^ Finally, extending TAVR to non-surgical centres will not necessarily reduce the waiting-list mortality rate, especially since the patients who die are not invariably those at highest risk. It is therefore essential to elucidate the causes of these deaths by focusing on the risk factors identified in our article.

### Study limitations

This study has several limitations. First, the design reduces external validity and may introduce selection bias. Additionally, the chronological difference in enrolment periods for the TAVR and deceased cohorts affects cumulative incidence results due to over-representation of patients who died after 2019. Second, comorbid patients may have extra-aortic cardiac lesions not necessarily attributable to AS and particularly stages 3–4. Third, an inherent limitation of Généreux staging is that patients in higher stages frequently have more comorbidities, leading to elevated rates of non-cardiac mortality and increased vulnerability to infections, trauma, and bleeding. Fourth, it is important to consider that patients currently in stage 2 may progress to more advanced stages while waiting. There is a risk of worsening outcomes for a larger group of patients. Although these constraints partially affect our comparison’s validity, registries remain essential to collect real-world data from unselected patient populations.

## Conclusion

This waiting period is critical, and delays exceeding 3 months appear to be detrimental. Findings from our study suggest that TAVR candidates at Généreux stages 3 and 4—both of which predict increased mortality—should also receive priority on the waiting list. Our study shows that current prioritization primarily focuses on LVEF, low cardiac output, or symptomatic presentation of AS. Going forward, it may be necessary to also prioritize patients who are classified as Généreux stages 3 or 4, those with elevated SPAP or right ventricular dysfunction, and those with coronary artery disease or renal insufficiency. In asymptomatic patients, we highlight the importance of incorporating the assessment of myocardial injury into cardiac staging, using both imaging and biomarkers. This comprehensive multimodal approach could enable more accurate and personalized triage, while minimizing the risk of clinical deterioration in patients initially at an early stage.

We also emphasize the need for external validation before the Généreux staging system can be operationally implemented for waitlist prioritization.

## Supplementary Material

qcag001_Supplementary_Data

## Data Availability

The data underlying this article will be shared on reasonable request to the corresponding author.

## Impact on daily practice

In this observational study, we used the Généreux stratification system for AS risk. Our results show that patients in stages 3 and 4 of Généreux classification are at higher risk of mortality while waiting for TAVR. Other major risk factors include right ventricular dysfunction and elevated pulmonary artery pressures, both of which significantly increase the likelihood of death.

Long waiting times lead to patient deaths. The longer patients wait, the more likely they are to advance to Généreux stages 3–4.
