# Correction to: Optimizing patient triage on the waiting list for transcatheter aortic valve replacement: the clinical utility of the cardiac damage staging system

**DOI:** 10.1093/ehjqcco/qcag048

**Published:** 2026-03-23

This is a correction to: Karen Boyer, Adrien Carmona, Francois Severac, Antonin Trimaille, Kévin Roulot, Shinnosuke Kikuchi, Marion Kibler, Patrick Ohlmann, Benjamin Marchandot, Olivier Morel, Optimizing patient triage on the waiting list for transcatheter aortic valve replacement: the clinical utility of the cardiac damage staging system, *European Heart Journal - Quality of Care and Clinical Outcomes*, Volume 12, Issue 4, June 2026, Pages 484–493, qcag001, https://doi.org/10.1093/ehjqcco/qcag001

The first name of the sixth co-author has been emended to read: “Shinnosuke Kikuchi”.

