# Supplementary material for: Optimizing patient triage on the waiting list for transcatheter aortic valve replacement: the clinical utility of the cardiac damage staging system
Source: Eur Heart J Qual Care Clin Outcomes. 2026 Mar 3;12(4):484–93. doi: 10.1093/ehjqcco/qcag001 (PMC13288729; doi:10.1093/ehjqcco/qcag001)
Supplement: qcag001_Supplementary_Data [file qcag001_supplementary_data.docx]

**Supplementary appendix**

| **Table S1. Descriptive echocardiographic analysis subjects by event** | | | | |
| --- | --- | --- | --- | --- |
|  | **Deaths** | **TAVR** | **Non-proceeding to TAVR** | **p (between death and TAVR)** |
| n | 98 | 1110 | 19 |  |
| **Echocardiographic parameters** | | | | |
| LVEF ([IQR] médian) | 60.00 [43.00, 60.00] | 56.00 [44.00, 64.00] | 56.00 [49.00, 61.50] | 0.875 |
| LVEDD ([IQR] médian) | 51.00 [45.00, 59.00] | 50.00 [45.00, 56.00] | 49.00 [40.75, 53.25] | 0.206 |
| LVTSD ([IQR] médian) | 33.00 [27.50, 44.50] | 34.00 [28.00, 41.00] | 35.00 [28.75, 40.25] | 0.722 |
| LV hypertrophy (indexed mass > 95/115 g/m^2^) (%) | 66 (76.7) | 837 (77.1) | 15 (88.2) | 1 |
| LA size (34 ml/m^2^ ou 20 cm^2^) (%) | 79 (84.0) | 935 (85.8) | 16 (84.2) | 0.646 |
| E/E’ > 14 (%) | 32 (43.2) | 308 (39.7) | 5 (38.5) | 0.62 |
| RVSF < 35% (%) | 29 (30.5) | 162 (14.9) | 2 (10.5) | < 0.001 |
| Moderate TR (%) | 6 (6.3) | 56 (5.1) | 1 (5.3) | 0.628 |
| Cardiac index (L/min/m^2^) ([median IQR]) | 2.70 [2.10, 3.50] | 3.00 [2.50, 3.60] | 2.90 [2.68, 4.30] | 0.024 |
| PI (median [IQR]) | 0.20 [0.17, 0.22] | 0.20 [0.17, 0.23] | 0.18 [0.16, 0.20] | 0.129 |
| AVA (median [IQR]) | 0.60 [0.45, 0.70] | 0.73 [0.60, 0.88] | 0.60 [0.54, 0.68] | < 0.001 |
| Mean gradient (median [IQR] médian) | 45.00 [33.00, 53.00] | 45.00 [39.00, 55.00] | 50.00 [43.50, 59.75] | 0.089 |
| SPAP > 60 mmHg (%) | 30 (31.6) | 125 (13.1) | 2 (11.1) | < 0.001 |
| LV hypertrophy (g) ([IQR] médian) | 130.00 [102.00, 157.00] | 128.00 [107.00, 156.00] | 128.50 [108.25, 148.50] | 0.895 |
| Indexed Systolic Ejection Volume ([IQR] médian) | 39.00 [29.33, 47.00] | 41.00 [33.00, 49.88] | 37.70 [32.25, 44.25] | 0.024 |
| MR (%) |  |  |  | < 0.001 |
| 0 | 72 (74.2) | 582 (54.6) | 11 (57.9) |  |
| 1 | 25 (25.8) | 300 (28.2) | 6 (31.6) |  |
| 2 | 0 (0.0) | 171 (16.1) | 2 (10.5) |  |
| 3 | 0 (0.0) | 6 (0.6) | 0 (0.0) |  |
| 4 | 0 (0.0) | 6 (0.6) | 0 (0.0) |  |
| *Data represented by medians with IQR (interquartile range) or percentages (%) with n the number of subjects ; AVA : Aortic Valve Surface, LA : Left Atrium, LV : Left Ventricle, LVEF : Left Ventricular Ejection Fraction, LVEDD : Left Ventricular End Diastolic Diameter, LVTSD : Left Ventricular Telesystolic Diameter, MR : Mitral Regurgitation, PI : Permeability Index, RVSF : Right Ventricular Shortening Fraction, SPAP : Systolic Pulmonary Arterial Pressure, TAVI : Transcatheter Aortic Valve Implantation, TAVR : Transcatheter Aortique Valve Replacement, TR : Tricuspid Regurgitation.* | | | | |

| **Table S2. Cause of death on waiting list (n=98)** |  |
| --- | --- |
| **Cause of death** | n (%) |
| **Cardiovascular death** | 47 (48) |
| Sudden death | 10 |
| Heart Failure or cardiogenic shock | 30 |
| Stroke | 1 |
| Aortic dissection | 4 |
| Tamponade | 1 |
| Acute lower limb ischemia | 1 |
| **Non-cardiovascular death** | 11 (11%) |
| **Unknown** | 40 (40%) |
